# *Helicobacter pylori* Neutrophil-Activating Protein Directly Interacts with and Activates Toll-like Receptor 2 to Induce the Secretion of Interleukin-8 from Neutrophils and ATRA-Induced Differentiated HL-60 Cells

**DOI:** 10.3390/ijms222111560

**Published:** 2021-10-26

**Authors:** Shao-Hsuan Wen, Zhi-Wei Hong, Chung-Chu Chen, Han-Wen Chang, Hua-Wen Fu

**Affiliations:** 1 Institute of Molecular and Cellular Biology, National Tsing Hua University, Hsinchu 30013, Taiwan; wen.shaohsuan@gmail.com (S.-H.W.); a76062908@gmail.com (Z.-W.H.); 2 Department of Internal Medicine, Division of Hepatology & Gastroenterology, Mackay Memorial Hospital, Hsinchu 30055, Taiwan; a4059@icloud.com; 3 Teaching Center of Natural Science, Minghsin University of Science and Technology, Hsinchu 30401, Taiwan; 4 Department of Life Science, National Tsing Hua University, Hsinchu 30013, Taiwan

**Keywords:** *Helicobacter pylori* neutrophil-activating protein, HP-NAP, TLR2, HL-60 cells, neutrophils, IL-8, PTX, ROS

## Abstract

*Helicobacter pylori* neutrophil-activating protein (HP-NAP)-induced production of reactive oxygen species (ROS) by neutrophils and monocytes is regulated by pertussis toxin (PTX)-sensitive G proteins, whereas HP-NAP-induced cytokine secretion by monocytes is mediated by Toll-like receptor 2 (TLR2). However, it is unclear whether TLR2 participates in HP-NAP-induced cytokine secretion by neutrophils. Here, all-trans retinoic acid (ATRA)-induced differentiated HL-60 cells were first employed as a neutrophil model to investigate the molecular mechanisms underlying neutrophil responses to HP-NAP. HP-NAP-induced ROS production in ATRA-induced differentiated HL-60 cells is mediated by the PTX-sensitive heterotrimeric G protein-dependent activation of extracellular signal-regulated kinase 1/2 and p38-mitogen-activated protein kinase, which is consistent with the findings reported for human neutrophils. Next, whether TLR2 participated in HP-NAP-induced secretion of interleukin-8 (IL-8) was investigated in neutrophils and ATRA-induced differentiated HL-60 cells. In both cells, TLR2 participated in HP-NAP-induced IL-8 secretion but not HP-NAP-induced ROS production. Interestingly, PTX-sensitive G proteins also contributed to the HP-NAP-induced secretion of IL-8 from neutrophils and the differentiated HL-60 cells. Our ELISA-based binding assay further revealed the competitive binding of Pam_3_CSK_4_, a TLR2 agonist, and HP-NAP to TLR2, which suggests the presence of specific and direct interactions between HP-NAP and TLR2. Thus, HP-NAP directly interacts with and activates TLR2 to induce IL-8 secretion in neutrophils and ATRA-induced differentiated HL-60 cells.

## 1. Introduction

*Helicobacter pylori* (*H. pylori*), a Gram-negative and microaerophilic bacterium, is found in the gastric mucosa of humans. *H. pylori* infects over half of the human population and its infection causes various gastroduodenal diseases including chronic gastritis and peptic ulcers [1]. Chronic gastric inflammation caused by *H. pylori* infection leads to neutrophil infiltration of the gastric mucosa [2,3]. The extent of neutrophil infiltration is correlated to the degree of mucosal damage in patients with *H. pylori* infection [4]. Neutrophil infiltration of the infection site of *H. pylori* can be attributed to the bacterial factors with chemotactic properties specific to neutrophils. *H. pylori* neutrophil-activating protein (HP-NAP), a key virulence factor of *H. pylori*, plays such a role because of its capability to attract and activate neutrophils.

HP-NAP is a spherical dodecamer consisting of ∼17 kDa identical subunits with a four-helix bundle structure [5,6]. This protein was first isolated from *H. pylori* water extracts because of its ability to induce neutrophil adhesion to endothelial cells and stimulate neutrophils to produce reactive oxygen species (ROS) [7]. HP-NAP also stimulates neutrophils to secrete myeloperoxidase, pro-inflammatory cytokines, and chemokines, such as interleukin-8 (IL-8) [8,9,10]. Once being activated by HP-NAP, the majority of neutrophils rapidly migrate to the infection sites and serve as sources of chemokines to recruit and activate additional neutrophils, as well as monocytes, dendritic cells, mast cells and lymphocytes, thereby generating a peculiar cytokine milieu at the infection site [9,11]. The ROS and cytokines, which are produced by neutrophils and other immune cells in response to HP-NAP, act as proinflammatory signals to induce the inflammation and damage of the gastric mucosa. This evidence suggests that HP-NAP plays a role in the immune pathogenesis of an *H. pylori* infection.

HP-NAP-induced ROS production by neutrophils is mediated by a pertussis toxin (PTX)-sensitive G protein-coupled receptor (GPCR) [8]. The intracellular signaling molecules, including the Src family of tyrosine kinase, phosphatidylinositol 3-kinase (PI3K), extracellular signal-regulated kinase 1/2 (ERK1/2), and p38-mitogen-activated protein kinase (p38-MAPK) are all involved in this event [8,12]. These signaling molecules promote the activation of nicotinamide adenine dinucleotide phosphate hydrogen (NADPH)-oxidase on the neutrophil membrane to generate ROS [8]. PTX-sensitive heterotrimeric G proteins also participate in HP-NAP-induced adhesion and chemotaxis of neutrophils [12]. In addition to being an agonist of PTX-sensitive GPCR, HP-NAP is a ligand of Toll-like receptor 2 (TLR2) [13]. The evidence comes from a study that showed HP-NAP inducing the activation of the nuclear factor kappa-light-chain-enhancer of activated B cells (NF-*κ*B) in HEK293 cells that were overexpressing TLR2, but not other TLRs, as assessed by the luciferase reporter assay [13]. It has also been reported that TLR2 mediates the HP-NAP-induced secretion of IL-6 in adherent splenocytes [14]. However, it is unclear whether TLR2 mediates the HP-NAP-induced neutrophil responses. One study showed that an enhanced expression of IL-8 in human neutrophils in response to *H. pylori* was suppressed by the pretreatment with TLR2-neutralizing antibody [15], indicating the involvement of TLR2 in the IL-8 production by neutrophils during an *H. pylori* infection. Because HP-NAP induces neutrophils to express and secrete IL-8 [9], TLR2 might mediate the HP-NAP-induced secretion of IL-8 by neutrophils.

Neutrophils are the key components of innate immune systems and the front line of the cellular defense against infection [16]. Their functions are regulated by a variety of cell-surface receptors, which recognize the invading microorganisms and sense the inflammatory environment [17,18]. Neutrophil responses that are induced by HP-NAP occur via the activation of its receptors and, subsequently, the complex signaling pathways. Because the primary neutrophils are very fragile and easily activated short-lived cells with donor variability [19,20], a suitable cell model for the study of the cellular mechanisms underlying neutrophil responses to HP-NAP is necessary. Human promyelocytic leukemia cell line HL-60 is the most commonly used cell line in neutrophil research [21]. HL-60 cells can be differentiated into neutrophil-like cells using all-trans retinoic acid (ATRA) or dimethyl sulfoxide (DMSO) [22,23]. Although differentiated HL-60 cells have been used to study various neutrophil functions, such as ROS production, chemotaxis, and NETosis [24,25], an appropriate differentiation condition for the conversion of HL-60 cells into the neutrophil-like cells that respond to HP-NAP needs to be determined.

In this study, we have first established a neutrophil-like model cell line that responds to HP-NAP by characterizing the ability of the differentiated HL-60 cells to produce ROS upon the treatment with HP-NAP and its associated signaling pathways. We then investigated whether TLR2 and PTX-sensitive G proteins participate in HP-NAP-induced IL-8 secretion by neutrophils and the differentiated HL-60 neutrophil-like cells. Finally, we examined whether HP-NAP directly and specifically interacts with TLR2.

## 2. Results

### 2.1. ATRA-Induced Differentiated HL-60 Cells as a Neutrophil Model to Study HP-NAP-Induced ROS Production

**Figure 1:**
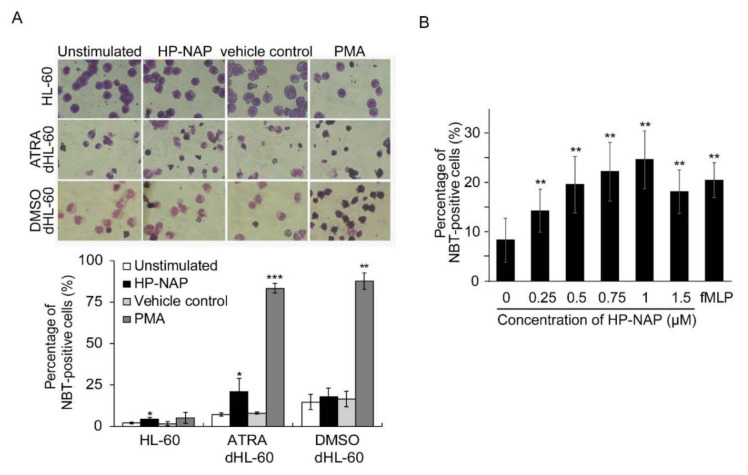
Induction of ROS production by HP-NAP in ATRA-induced differentiated HL-60 cells but not in DMSO-induced differentiated HL-60 cells. (**A**) Effect of HP-NAP on ROS production in undifferentiated and ATRA- and DMSO-induced differentiated HL-60 cells. HL-60 cells were treated with 1 μM ATRA or 1.25% DMSO for 4 days to induce the differentiation. Undifferentiated HL-60 cells and differentiated HL-60 (dHL-60) cells at a density of 1 × 10^6^ cells/mL were stimulated with 1 μM HP-NAP, D-PBS, pH 7.2, as the unstimulated control, 0.32 μM PMA, or DMSO as the vehicle control for PMA, at 37 °C for 30 min. ROS produced by these cells were measured by NBT reduction assay as described in Materials and Methods and expressed as percentage of NBT-positive cells. Data are expressed as mean ± SD of at least three independent experiments. (**B**) Dose-response effect of HP-NAP on ROS production by ATRA-induced differentiated HL-60 cells. ATRA-induced differentiated HL-60 cells at a density of 1 × 10^6^ cells/mL were stimulated with the indicated concentrations of HP-NAP in the range of 0.25 μM to 1.5 μM or 1 μM fMLP as a positive control at 37 °C for 30 min. ROS production was determined by NBT reduction assay as described in A. Data are expressed as mean ± SD of six independent experiments. *: *p* value < 0.05, **: *p* value < 0.01, ***: *p* value < 0.001 as compared with the unstimulated control for HP-NAP-stimulated cells or the vehicle control for PMA-stimulated cells.

The human promyelocytic HL-60 cell line can be differentiated in vitro into neutrophils by retinoic acid or DMSO [22,23]. After differentiation, neutrophil-like HL-60 cells express most of the components of NADPH oxidase and are capable of ROS production [26,27]. To determine whether HP-NAP induces ROS production in HL-60 differentiated neutrophil-like cells, we treated HL-60 cells with 1 μM ATRA and 1.25% DMSO for four days and examined if HP-NAP induces an increase in the number of ROS-producing cells in these two differentiated HL-60 cells by using a nitro blue tetrazolium (NBT) reduction assay. Both ATRA and DMSO induced the differentiation of HL-60 cells into neutrophils, as assessed by the morphological change and the expression of CD11b (Supplementary Table S1 and Supplementary Figures S1 and S2). In ATRA-induced differentiated HL-60 cells, HP-NAP induced a significant increase in the number of ROS-producing cells from 7 ± 1% to 23 ± 8%, whereas in DMSO-induced differentiated HL-60 cells, HP-NAP induced no significant increase in the number of ROS-producing cells from 14 ± 4% to 17 ± 5% (Figure 1A). Under the unstimulated condition, a higher number of ROS-producing cells was found in the DMSO-induced differentiated HL-60 cells than in the ATRA-induced differentiated HL-60 cells (Figure 1A). The number of ROS-producing cells noticeably increased in both of the differentiated HL-60 cells that were stimulated by phorbol 12-myristate 13-acetate (PMA), suggesting that both of the differentiated HL-60 cells are neutrophil-like. Less than 10% of undifferentiated HL-60 cells were capable of reducing NBT after stimulation by HP-NAP or PMA (Figure 1A). We then measured the production of ROS from ATRA-induced differentiated HL-60 cells that were stimulated with increasing concentrations of HP-NAP in the range of 0.25 μM to 1.5 μM. After stimulation of the ATRA-induced differentiated HL-60 cells by HP-NAP, the number of ROS-producing cells was significantly increased in a dose-dependent manner and reached a maximum at the concentration of 1 μM of HP-NAP (Figure 1B). These results indicate that HP-NAP is able to induce ROS production in ATRA-induced differentiated HL-60 cells. ATRA-induced differentiated HL-60 cells were chosen as a neutrophil model to investigate the cell signal responses that are triggered by HP-NAP.

### 2.2. Involvement of PTX-Sensitive Heterotrimeric G Proteins in HP-NAP-Induced ROS Production in ATRA-Induced Differentiated HL-60 Cells

**Figure 2:**
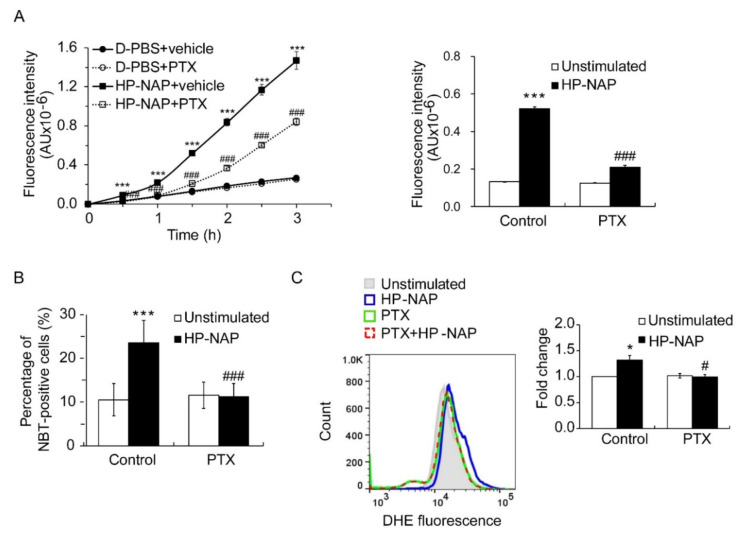
PTX-dependent inhibition of HP-NAP-induced ROS production in neutrophils and ATRA-induced differentiated HL-60 cells. (**A**) The inhibitory effect of PTX on HP-NAP-induced ROS production by neutrophils. Neutrophils at a density of 2 × 10^6^ cells/mL were pretreated with 100 ng/mL PTX or the vehicle control at 37 °C for 3 h and followed by the stimulation with 1 μM HP-NAP or D-PBS, pH 7.2, as the unstimulated control at 37 °C for the indicated time. ROS produced by neutrophils was measured by H_2_DCF-DA-derived fluorescence assay as described in Materials and Methods. Data from neutrophils treated with HP-NAP for 1.5 h are shown as bar graph and expressed as mean ± SD of four independent experiments in triplicate. (**B**) The inhibitory effect of PTX on the number of ROS-producing cells in ATRA-induced differentiated HL-60 cells induced by HP-NAP. ATRA-differentiated HL-60 cells at a density of 4 × 10^6^ cells/mL were pretreated with 100 ng/mL PTX or the vehicle control at 37 °C for 16 h and followed by the stimulation with 1 μM HP-NAP or D-PBS, pH 7.2, as the unstimulated control at 37 °C for 30 min. The number of ROS-producing cells was determined by NBT reduction assay as described in Figure 1A. Data are expressed as mean ± SD of six independent experiments. (**C**) The inhibitory effect of PTX on the level of ROS production in ATRA-induced differentiated HL-60 cells induced by HP-NAP. ATRA-induced differentiated HL-60 cells at a density of 4 × 10^6^ cells/mL were pretreated with 100 ng/mL PTX or the vehicle control at 37 °C for 16 h and followed by the stimulation with 1 μM HP-NAP or D-PBS, pH 7.2, as the unstimulated control at 37 °C for 30 min. The level of ROS production was measured by monitoring the cell-derived DHE fluorescence using flow cytometry as described in Materials and Methods. The representative histogram is shown in the left panel. Data in the right panel are expressed as the fold change of the mean fluorescence intensity (MFI) by defining the MFI of DHE-derived fluorescence in the unstimulated control cells as 1 and as mean ± S.D. of three independent experiments. *: *p* value < 0.05, ***: *p* value < 0.001 as compared with unstimulated cells. #: *p* value < 0.05, ###: *p* value < 0.001 as compared with HP-NAP-stimulated cells.

HP-NAP-induced ROS production is mediated by PTX-sensitive heterotrimeric G proteins in human neutrophils [8]. To confirm this finding, we first employed a redox-sensitive fluorescent dye, 2’,7’-dichlorofluorescin diacetate (H_2_DCF-DA), to detect the production of ROS by neutrophils that were isolated from human peripheral blood. After stimulation with HP-NAP, the neutrophils produced ROS in a time-dependent manner within 3 h (Figure 2A). HP-NAP-induced ROS production was markedly suppressed by the pretreatment of neutrophils with PTX (Figure 2A). This phenomenon was most evident when neutrophils were stimulated with HP-NAP for 1.5 h (Figure 2A). At this time point, the pretreatment of neutrophils with PTX markedly suppressed the increase of HP-NAP-induced ROS production from 4-fold to about 2.7-fold (Figure 2A). This result indicates that HP-NAP-induced ROS production in human neutrophils is mediated by a PTX-sensitive pathway, which is consistent with the previous report [8]. We next investigated whether PTX-sensitive heterotrimeric G proteins mediated HP-NAP-induced ROS production in ATRA-induced differentiated HL-60 cells. In cells that were stimulated with HP-NAP, the number of ROS-producing cells was significantly increased from 10.5 ± 4% to 23.5 ± 5% and the amount of ROS production was significantly increased to 1.38-fold over the basal level, as determined by an NBT reduction assay and a dihydroethidium (DHE) fluorescence assay, respectively (Figure 2B,C). The increase in ROS production induced by HP-NAP was significantly inhibited in cells that were pretreated with PTX (Figure 2B,C). Thus, the HP-NAP-induced production of ROS is mediated by PTX-sensitive heterotrimeric G proteins in ATRA-induced differentiated HL-60 cells.

### 2.3. HP-NAP-Induced PTX-Sensitive Heterotrimeric G Protein-Dependent Activation of ERK1/2 and p38-MAPK to Elicit ROS Production in ATRA-Induced Differentiated HL-60 Cells

**Figure 3:**
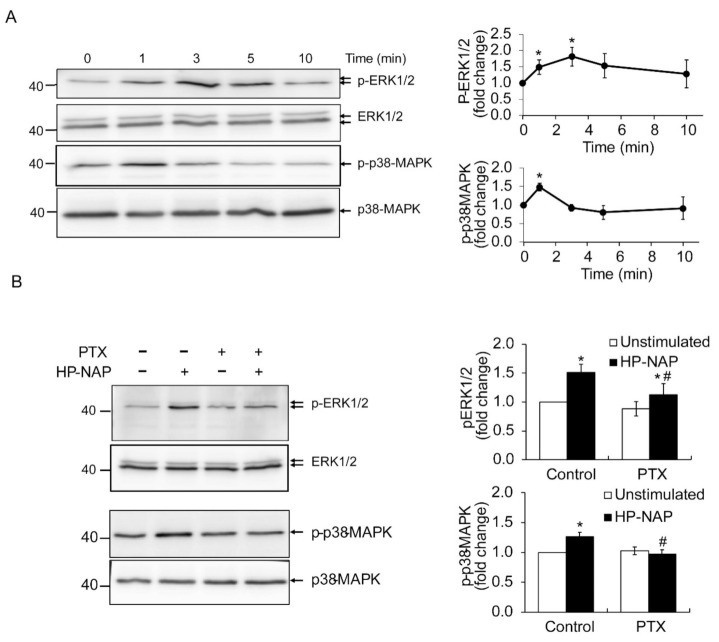
Involvement of PTX-sensitive G-proteins in HP-NAP-induced activation of ERK1/2 and p38-MAPK in ATRA-induced differentiated HL-60 cells. (**A**) HP-NAP-induced phosphorylation of ERK1/2 and p38-MAPK in ATRA-induced differentiated HL-60 cells. ATRA-induced differentiated HL-60 cells at a density of 2.5 × 10^6^ cell/mL were stimulated with 1 μM HP-NAP or D-PBS, pH 7.2, as the unstimulated control at 37 °C for the indicated time. The cells were lysed and whole cell lysates were applied to immunoblot analysis for phospho-ERK1/2, ERK1/2, phospho-p38-MAPK, and p38-MAPK. Quantitative data are expressed as the fold change by defining the amounts of the phosphorylated proteins in unstimulated control cells as 1 and as mean ± SD of at least three independent experiments. (**B**) Effect of PTX on HP-NAP-induced phosphorylation of ERK1/2 and p38-MAPK in ATRA-induced differentiated HL-60 cells. ATRA-induced differentiated HL-60 cells at a density of 5 × 10^6^ cell/mL were pretreated with 100 ng/mL PTX or the vehicle control at 37 °C for 16 h and followed by the stimulation with 1 μM HP-NAP or D-PBS, pH 7.2, as the unstimulated control at 37 °C for 3 min to examine the phosphorylation of ERK1/2 or 1 min to examine the phosphorylation of p38-MAPK. The cells were lysed and whole cell lysates were applied to immunoblot analysis as described in A. Quantitative data are expressed as the fold change described in A and as mean ± SD of three independent experiments. *: *p* value < 0.05 as compared with unstimulated cells with in each group; #: *p* value < 0.05 as compared with HP-NAP-stimulated control cells.

**Figure 4:**
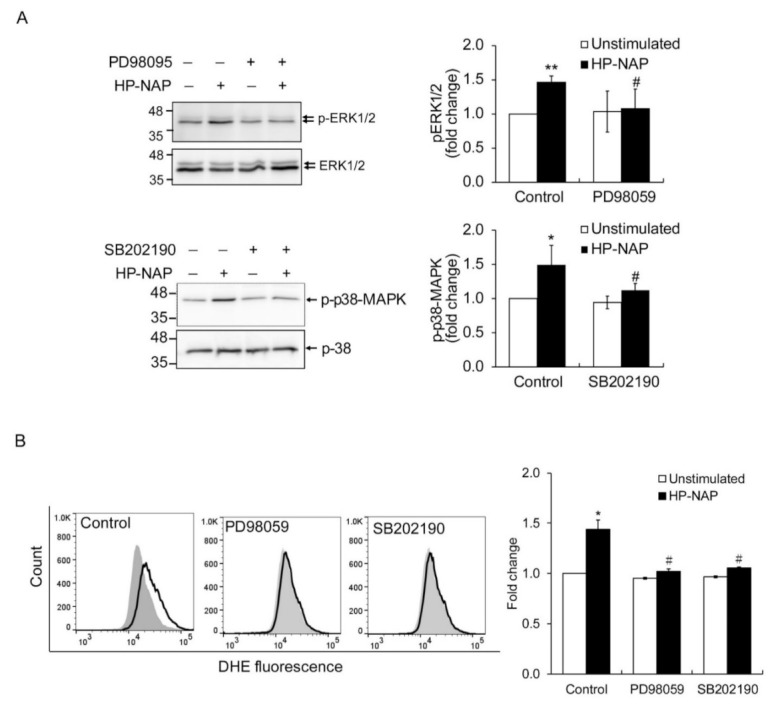
The effect of MAPK inhibitors on MAPK phosphorylation and ROS production in ATRA-induced differentiated HL-60 cells in response to HP-NAP. (**A**) The inhibitory effect of MAPK inhibitors on HP-NAP-induced MAPK phosphorylation in ATRA-induced differentiated HL-60 cells. ATRA-induced differentiated HL-60 cells at a density of 5 × 10^6^ cell/mL were pretreated with 10 μM PB98059, 5 μM SB202190, or the vehicle control at 37 °C for 1 h and followed by the stimulation with 1 μM HP-NAP or D-PBS, pH 7.2, as the unstimulated control for 3 min to detect the phosphorylation of ERK1/2 or 1 min to detect the phosphorylation of p38-MAPK. Cells were lysed and whole cell lysates were applied to immunoblot analysis as described in Figure 3A. Quantitative data are expressed as the fold change as described in Figure 3A and as mean ± SD of four independent experiments. (**B**) The effect of MAPK inhibitors on HP-NAP-induced ROS production in ATRA-induced differentiated HL-60 cells. ATRA-induced differentiated HL-60 cells at a density of 4 × 10^6^ cells/mL were pretreated with MAPK inhibitors or the vehicle control as described in A and followed by the stimulation with 1 μM HP-NAP or D-PBS, pH 7.2, as the unstimulated control at 37 °C for 30 min. ROS production was measured as DHE-derived fluorescence detected by flow cytometry as described in Figure 2C. Representative histograms are shown in the left panel. Gray filled histograms represent the unstimulated control cells and black open histograms represent the HP-NAP-stimulated cells. Data in the right panel are expressed as the fold change of MFI by defining the MFI of DHE-derived fluorescence in the unstimulated control cells as 1 and as mean ± SD of three independent experiments. *: *p* value < 0.05, **: *p* value < 0.01 as compared with the unstimulated cells in each group; #: *p* value < 0.05 as compared with HP-NAP-stimulated cells in the control group.

In human neutrophils, HP-NAP activates PTX-sensitive heterotrimeric G proteins and then induces the phosphorylation of ERK1/2 and p38-MAPK to produce ROS [12]. To further determine if these HP-NAP-mediated signaling events also occur in ATRA-induced differentiated HL-60 cells, we investigated whether HP-NAP induced the activation of PTX-sensitive heterotrimeric G proteins to produce ROS via phosphorylation of ERK1/2 and p38-MAPK. First, we performed a time-course experiment to examine whether HP-NAP induced the phosphorylation of ERK1/2 and p38-MAPK in ATRA-induced differentiated HL-60 cells. As shown in Figure 3A, the stimulation of ATRA-induced differentiated HL-60 cells with HP-NAP resulted in a rapid and significant phosphorylation of ERK1/2 and p38-MAPK. The phosphorylation of ERK1/2 reached a maximum within 3 min of stimulation and the phosphorylation of p38-MAPK reached a maximum within 1 min of stimulation (Figure 3A). Next, we examined whether the PTX-sensitive heterotrimeric G proteins were involved in the HP-NAP-induced phosphorylation of ERK1/2 and p38-MAPK in ATRA-induced differentiated HL-60 cells. As shown in Figure 3B, pretreatment of the cells with PTX significantly suppressed the phosphorylation of ERK1/2 and p38-MAPK. These findings indicate an involvement of PTX-sensitive heterotrimeric G proteins in the HP-NAP-induced activation of ERK1/2 and p38-MAPK in ATRA-induced differentiated HL-60 cells. The possible participation of ERK1/2 and p38-MAPK in HP-NAP-induced ROS production was further explored in ATRA-induced differentiated HL-60 cells by using the inhibitors against MEK1/2, the upstream molecule of ERK1/2, and p38-MAPK. Although the HP-NAP-induced phosphorylation of ERK1/2 and p38-MAPK was significantly suppressed in the ATRA-induced differentiated HL-60 cells that were pretreated with PD98059, a MEK1/2 inhibitor, and SB202190, a p38-MAPK inhibitor, respectively (Figure 4A), the HP-NAP-induced production of ROS was almost completely suppressed in the cells that were pretreated with either PD98059 or SB202190 (Figure 4B). Taken together, these results indicate that the HP-NAP-induced production of ROS is mediated by the PTX-sensitive heterotrimeric G protein-dependent phosphorylation of ERK1/2 and p38-MAPK in ATRA-induced differentiated HL-60 cells. The molecular mechanism by which HP-NAP induces ROS production in ATRA-induced differentiated HL-60 cells is similar to that of neutrophils. This finding further supports the idea that ATRA-induced differentiated HL-60 cells serve as a suitable model for the study of the molecular mechanisms underlying the neutrophil responses to HP-NAP.

### 2.4. TLR2-Independent ROS Production in Neutrophils and ATRA-Induced Differentiated HL-60 Cells Induced by HP-NAP

**Figure 5:**
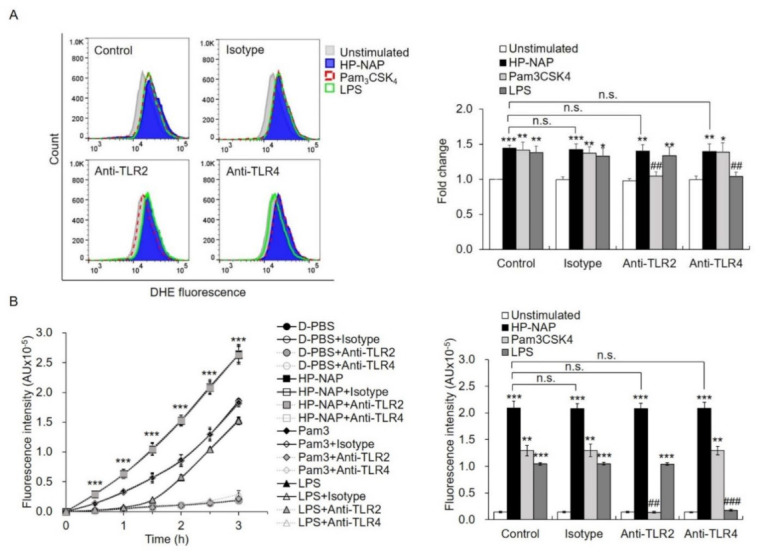
The effect of TLR2-neutralizing antibodies on HP-NAP-induced ROS production by ATRA-induced differentiated HL-60 cells and neutrophils. (**A**) TLR2-independent ROS production in ATRA-induced differentiated HL-60 cells induced by HP-NAP. ATRA-induced differentiated HL-60 cells at a density of 4 × 10^6^ cell/mL were pretreated with 10 μg/mL of anti-TLR2, anti-TLR4, or IgG2a isotype antibodies at 37 °C for 30 min and followed by the stimulation with 1 μM HP-NAP, 1 μg/mL Pam_3_CSK_4_, 10 μg/mL LPS, or D-PBS, pH 7.2, as the unstimulated control at 37 °C for 30 min. ROS production was measured as DHE-derived fluorescence by flow cytometry as described in Figure 2C. Representative histograms are shown in the left panel. Data in the right panel are expressed as the fold change as described in Figure 2C and as mean ± SD of three independent experiments. (**B**) TLR2-independent ROS production in neutrophils induced by HP-NAP. Neutrophils at a density of 2 × 10^6^ cell/mL were pretreated with 10 μg/mL of anti-TLR2, anti-TLR4 or IgG2a isotype antibodies at 37 °C for 30 min and followed by the stimulation with 1 μM HP-NAP, 1 μg/mL Pam_3_CSK_4_, 10 μg/mL LPS or D-PBS, pH 7.2, as the unstimulated control for the indicated time. ROS production by neutrophils was determined by H_2_DCF-DA-derived derived fluorescence assay as described in Figure 2A. Data from neutrophils stimulated with HP-NAP for 2.5 h are shown as bar graph and expressed as mean ± SD of four independent experiments in duplicate. *: *p* value < 0.05, **: *p* value < 0.01, ***: *p* value < 0.001 as compared with the unstimulated cells. ##: *p* value < 0.01, ###: *p* value < 0.001 as compared with HP-NAP-stimulated cells in the isotype control group. n.s.: non-significant.

It has been reported that the activation of TLR2 by various bacterial components is able to induce ROS production in neutrophils, thereby triggering downstream immune responses [28,29]. However, it is unclear whether TLR2 is involved in the HP-NAP-induced ROS production in neutrophils. To investigate whether TLR2 participates in the HP-NAP-induced production of ROS in ATRA-induced differentiated HL-60 cells and neutrophils, we examined whether pretreatment of these cells with a TLR2-neutralizing antibody is able to inhibit the ROS production induced by HP-NAP. In this experiment, the cells were also pretreated with a TLR4-neutralizing antibody as a control due to the fact that both TLR2 and TLR4 are expressed on the surface of neutrophils and mediate common neutrophil responses to various invading pathogens through similar downstream signaling pathways [30,31]. We found that pretreatment with either the TLR2-neutralizing antibody or the TLR4-neutralizing antibody did not inhibit HP-NAP-induced production of ROS in ATRA-induced differentiated HL-60 cells, as measured by DHE-derived fluorescence (Figure 5A). However, the ROS production in ATRA-induced differentiated HL-60 cells induced by Pam_3_CSK_4_, a TLR2 agonist, and lipopolysaccharide (LPS), a TLR4 agonist, was significantly inhibited by the TLR2-neutralizing antibody and the TLR4-neutralizing antibody, respectively (Figure 5A). In cells that were pretreated with the isotype control antibody, the production of ROS that was induced by HP-NAP, Pam_3_CSK_4_, and LPS was not affected (Figure 5A). These results indicate that TLR2 does not participate in the HP-NAP-induced ROS production in ATRA-induced differentiated HL-60 cells. The results also confirmed the specificity of antibodies and excluded the possibility of an endotoxin contamination from the purified HP-NAP. Similar experiments were performed with human neutrophils. HP-NAP, Pam_3_CSK_4_, and LPS all induced ROS production in neutrophils in a time-dependent manner, as measured by H_2_DCF-DA-derived fluorescence (Figure 5B). In neutrophils, the ROS production that was induced by Pam_3_CSK_4_ and LPS was significantly inhibited by the TLR2-neutralizing antibody and the TLR4-neutralizing antibody, respectively, whereas the ROS production induced by HP-NAP was not inhibited by either the TLR2-neutralizing antibody or the TLR4-neutralizing antibody (Figure 5B). These results indicate that TLR2 does not participate in the HP-NAP-induced ROS production in human neutrophils. Taken together, the HP-NAP-induced ROS productions in both ATRA-induced differentiated HL-60 cells and human neutrophils is independent of TLR2.

### 2.5. Involvement of TLR2 and PTX-Sensitive Heterotrimeric G Proteins in HP-NAP-Induced IL-8 Secretion by Neutrophils and ATRA-Induced Differentiated HL-60 Cells

**Figure 6:**
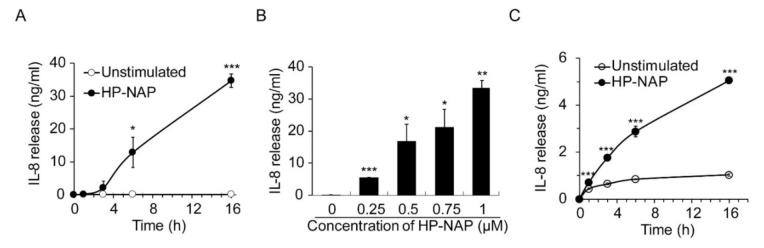
Time- and dose-dependent induction of IL-8 secretion by HP-NAP. (**A**,**B**) Time- and dose-dependent secretion of IL-8 from ATRA-induced differentiated HL-60 cells induced by HP-NAP. ATRA-induced differentiated HL-60 cells at a density of 1 × 10^6^ cells/mL were stimulated with 1 μM HP-NAP or D-PBS, pH 7.2, as the unstimulated control at 37 °C for the indicated time (**A**) or stimulated with the indicated concentrations of HP-NAP (**B**) at 37 °C for 16 h. The amount of IL-8 release was measured from the culture supernatants by ELISA. Data are expressed as mean ± SD of four and three independent experiments for A and B, respectively. (**C**) Time-dependent secretion of IL-8 from neutrophils induced by HP-NAP. Neutrophils at a density of 2.5 × 10^6^ cells/mL were treated with 1 μM HP-NAP and D-PBS, pH 7.2, as the unstimulated control at 37 °C for the indicated time. The amount of IL-8 release was determined as described in (**A**,**B**). Data are expressed as mean ± SD of four independent experiments. *: *p* < 0.05, **: *p* value < 0.01, ***: *p* < 0.001 as compared with the unstimulated cells.

**Figure 7:**
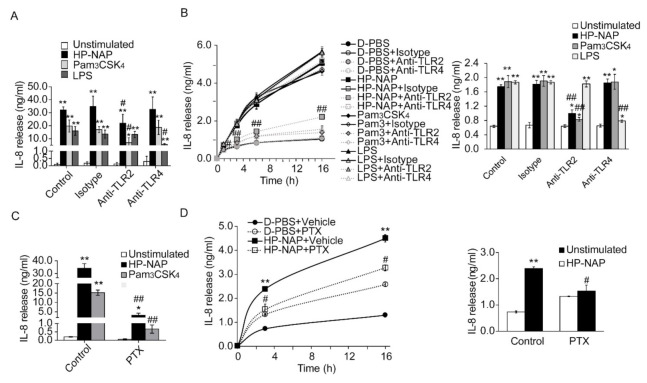
Involvement of TLR2 and PTX-sensitive heterotrimeric G proteins in HP-NAP-induced secretion of IL-8 by ATRA-induced differentiated HL-60 cells and neutrophils. (**A**) TLR2-dependent secretion of IL-8 from ATRA-induced differentiated HL-60 cells induced by HP-NAP. ATRA-induced differentiated HL-60 cells at a density of 2 × 10^6^ cells/mL were pretreated with 10 μg/mL of anti-TLR2, anti-TLR4, or IgG2a isotype antibodies at 37 °C for 30 min and followed by the stimulation with 1 μM HP-NAP, 10 ng/mL Pam_3_CSK_4_, 10 μg/mL LPS or D-PBS, pH 7.2, as the unstimulated control at 37 °C for 16 h. The amount of IL-8 release was determined as described in Figure 6. Data are expressed as mean ± SD of four independent experiments. (**B**) TLR2-dependent secretion of IL-8 from neutrophils induced by HP-NAP. Neutrophils at a density of 5 × 10^6^ cells/mL were pretreated with 10 μg/mL of anti-TLR2, anti-TLR4, or IgG2a isotype antibodies at 37 °C for 30 min and followed by the stimulation with 1 μM HP-NAP, 1 μg/mL Pam_3_CSK_4_, 10 μg/mL LPS or D-PBS, pH 7.2, as the unstimulated control at 37 °C for the indicated time. The amount of IL-8 release was determined as described in A. Data from neutrophils stimulated with HP-NAP for 3 h are shown as the bar graph and expressed as mean ± SD of four independent experiments. (**C**) PTX-sensitive secretion of IL-8 from ATRA-induced differentiated HL-60 cells induced by HP-NAP and Pam_3_CSK_4_. ATRA-induced differentiated HL-60 cells at a density of 2 × 10^6^ cells/mL were pretreated with 100 ng/mL PTX or the vehicle control at 37 °C for 16 h and followed by the stimulation with 1 μM HP-NAP, 10 ng/mL Pam_3_CSK_4_, or D-PBS as unstimulated control at 37 °C for 16 h. The amount of IL-8 release was determined as described in A. Data are expressed as mean ± SD of three independent experiments. (**D**) PTX-sensitive secretion of IL-8 from neutrophils induced by HP-NAP. Neutrophils at a density of 5 × 10^6^ cells/mL were pretreated with 100 ng/mL PTX or the vehicle control at 37 °C for 4 h and followed by the stimulation with 1 μM HP-NAP or D-PBS, pH 7.2, as the unstimulated control at 37 °C for the indicated time. The amount of IL-8 release was determined as described in A. Data from neutrophils stimulated with HP-NAP for 3 h are shown as the bar graph and expressed as mean ± SD of four independent experiments. *: *p* value < 0.05, **: *p* value < 0.01 as compared with unstimulated cells. #: *p*-value < 0.05, ##: *p* value < 0.01 as compared with stimulated cells in isotype control group or vehicle control group.

It has been reported that TLR2 mediates the HP-NAP-induced cytokine secretion from monocytes and splenocytes [13,14]. Although HP-NAP induces the secretion of IL-8 from neutrophils [9], it is unclear whether this is mediated by TLR2. Here, we first examined whether HP-NAP induced the secretion of IL-8 from ATRA-induced differentiated HL-60 cells. As shown in Figure 6A, the stimulation of ATRA-induced differentiated HL-60 cells with 1 μM of HP-NAP led to a time-dependent release of IL-8. HP-NAP-induced IL-8 release by these cells was dose-dependent at concentrations ranging from 0.25 μM to 1 μM (Figure 6B). Similarly, HP-NAP induced human neutrophils to release IL-8 in a time-dependent manner (Figure 6C). We then investigated whether TLR2 mediated the HP-NAP-induced secretion of IL-8 from ATRA-induced differentiated HL-60 cells and neutrophils by pretreating the cells with a TLR2-neutralizing antibody to inhibit TLR2 activity. The TLR4-neutralizing antibody was used as a control. Pretreatment of the ATRA-induced differentiated HL-60 cells with a TLR2-neutralizing antibody and a TLR4-neutralizing antibody specifically and significantly inhibited the IL-8 secretion that was induced by Pam_3_CSK_4_ and LPS, respectively (Figure 7A). Pretreatment of the ATRA-induced differentiated HL-60 cells with a TLR2-neutralizing antibody resulted in a 37% reduction in the IL-8 secretion induced by HP-NAP, whereas pretreatment with the IgG2a isotype control had no significant effect (Figure 7A). As for human neutrophils, pretreatment with a TLR2-neutralizing antibody resulted in a 45% reduction in the secretion of IL-8 after 3 h of stimulation with HP-NAP (Figure 7B). These results indicate that the HP-NAP-induced secretion of IL-8 from ATRA-induced differentiated HL-60 cells and neutrophils is partially mediated by TLR2. Because PTX-sensitive heterotrimeric G proteins are involved in many neutrophil responses that are induced by HP-NAP [8], we also investigated whether PTX-sensitive heterotrimeric G proteins participated in the HP-NAP-induced secretion of IL-8 by ATRA-induced differentiated HL-60 cells and neutrophils. In ATRA-induced differentiated HL-60 cells, pretreatment with PTX inhibited the HP-NAP-induced IL-8 secretion by 91%, whereas the same pretreatment also inhibited the Pam_3_CSK_4_-induced IL-8 secretion by 96% (Figure 7C). In human neutrophils, the time-dependent IL-8 secretion induced by HP-NAP was inhibited by the pretreatment of PTX (Figure 7D). This pretreatment resulted in a 36% reduction in IL-8 secretion by neutrophils after HP-NAP stimulation for 3 h (Figure 7D). Unexpectedly, pretreatment of PTX by itself stimulated neutrophils to secrete IL-8 (Figure 7D). It is possible that PTX induces the secretion of IL-8 from neutrophils as it does from endothelial cells [32]. Nevertheless, the inhibitory effect of PTX on the HP-NAP-induced secretion of IL-8 by neutrophils was still significant (Figure 7D). The results indicate that PTX-sensitive heterotrimeric G proteins might participate in the HP-NAP-induced secretion of IL-8 by ATRA-induced differentiated HL-60 cells and neutrophils. Taken together, both TLR2 and PTX-sensitive heterotrimeric G proteins are involved in the HP-NAP-induced IL-8 secretion by neutrophils and ATRA-induced differentiated HL-60 cells.

### 2.6. Specific Physical Interaction between HP-NAP and TLR2

**Figure 8:**
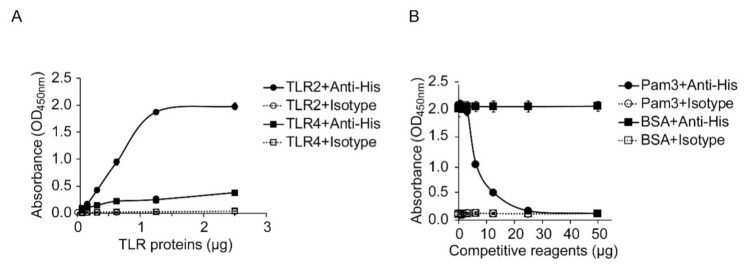
Specific interaction of HP-NAP with recombinant human TLR2 protein. (**A**) Binding of TLR2 to HP-NAP as determined by an ELISA-based solid-phase binding assay. The indicated amounts of recombinant human TLR2-10xHis and TLR4-10xHis proteins were incubated with 0.05 μg of HP-NAP coated on an ELISA plate. The binding of TLR2 and TLR4 to HP-NAP was detected by anti-His tag antibody or its isotype control antibody using the ELISA-based solid-phase binding assay as described in Materials and Methods. The results are presented as absorbance at OD_450 nm_. Data are expressed as mean ± SD of two independent experiments. (**B**) Specific binding of TLR2 to HP-NAP as determined by competitive binding with Pam_3_CSK_4_. The indicated amounts of Pam_3_CSK_4_ (Pam3) and BSA, as a negative control, were incubated with 0.625 μg of the recombinant human TLR2-10xHis protein at 4 °C overnight and then incubated with 0.05 μg of HP-NAP coated on an ELISA plate. The binding of TLR2 to HP-NAP was detected by the ELISA-based solid-phase binding assay as described in A. The results were presented as absorbance at OD_450 nm_. Data are expressed as mean ± SD of two independent experiments.

The finding that both TLR2 and PTX-sensitive heterotrimeric G proteins are involved in HP-NAP-induced IL-8 secretion by neutrophils prompts us to wonder whether TLR2 mediates this event through direct ligand-receptor interactions. Although it has been reported that HP-NAP is the ligand of TLR2 [13], no evidence supports a direct interaction between HP-NAP and TLR2. Here, we first performed an ELISA-based solid-phase binding assay modified from a previously described recombinant NAP-based ELISA [33] to examine whether HP-NAP directly interacts with TLR2. In this experiment, 0.05 μg of HP-NAP was pre-coated onto the wells of a 96-well plate, recombinant human TLR2-10xHis and TLR4-10xHis proteins, starting at 0.078125 μg and increasing in two-fold intervals up to 2.5 μg, were added to the wells, and then the two recombinant TLR proteins that were interacting with HP-NAP were detected with the anti-His tag antibody. We found that TLR2 bound to HP-NAP in a dose-dependent manner (Figure 8A). The binding curve appears to be sigmoidal, and the binding reached a plateau at the amount of 1.25 μg of TLR2 proteins (Figure 8A). The half-maximal binding of TLR2 to HP-NAP occurred at around 0.625 μg of TLR2 proteins (Figure 8A). Although the binding of TLR4 to HP-NAP appears to be dose-dependent, it was much weaker than the binding of TLR2 to HP-NAP (Figure 8A). Almost no detectable signal was found in the isotype control (Figure 8A). The binding of TLR4 to HP-NAP is presumably due to the non-specific interactions between the two proteins or between HP-NAP and the His tag that was fused to TLR4. These results indicate that HP-NAP directly interacts with TLR2. We next sought to further confirm that the interaction of TLR2 with HP-NAP was specific. Pam_3_CSK_4_, an agonist of TLR2, was employed to compete with HP-NAP for the binding of TLR2. In this experiment, an amount of 0.625 μg of TLR2 proteins was added to the surface-immobilized HP-NAP in the presence of Pam_3_CSK_4_, the amounts of which ranged from 0.78125 μg to 50 μg and were separated by increases at two-fold intervals. We found that Pam_3_CSK_4_ inhibited the binding of TLR2 to the immobilized HP-NAP with a half-maximal inhibitory concentration (IC_50_) of around 6.25 µg (Figure 8B). However, the binding of TLR2 to HP-NAP was not affected by the addition of up to 50 μg of bovine serum albumin (BSA) as a control (Figure 8B). These results indicate that there is a specific physical interaction between HP-NAP and TLR2.

## 3. Discussion

In this study, we provide the first evidence that HP-NAP directly interacts with TLR2. Upon being activated by HP-NAP, TLR2 mediates the secretion of IL-8, but not the production of ROS, by human neutrophils and the ATRA-induced differentiated HL-60 cells. Interestingly, PTX-sensitive heterotrimeric G proteins are involved in both IL-8 secretion and ROS production by neutrophils and ATRA-induced differentiated HL-60 cells in response to HP-NAP. In addition, we show that HP-NAP-induced ROS production in ATRA-induced differentiated HL-60 cells is mediated by the PTX-sensitive heterotrimeric G protein-dependent activation of ERK1/2 and p38-MAPK signaling pathways, which is consistent with the findings that were reported for human neutrophils [8,12]. Thus, ATRA-induced differentiated HL-60 cells can serve as a suitable model to study the molecular mechanisms underlying neutrophil responses to HP-NAP.

Neutrophils play a crucial role in host defense against invading microorganisms. TLRs are essential for neutrophils to recognize various microbial pathogens and induce host-innate responses [17,34]. TLR2, one of the most well-characterized cell-surface TLRs, modulates a wide range of neutrophil functions, such as the production of cytokine and chemokine, ROS production, degranulation, phagocytosis, chemotaxis, and the formation of neutrophil extracellular trap (NET) [17,28,29,34]. In this report, we show that TLR2 mediates the HP-NAP-induced secretion of IL-8 by neutrophils. Although one could argue that the inhibitory effect of the TLR2-neutralizing antibody on the HP-NAP-induced secretion of IL-8 by the differentiated HL-60 cells and neutrophils is due to the inhibition of TLR2 activation by endogenous factors resulting from the prolonged stimulation with HP-NAP for 16 h rather than by HP-NAP itself, the inhibitory effect of the TLR2-neutralizing antibody on IL-8 secretion from neutrophils by short-term stimulation with HP-NAP for 3 h is still significant. Most importantly, our ELISA-based solid-phase binding assay revealed the competitive binding of TLR2 between Pam_3_CSK_4_, a TLR2 agonist, and HP-NAP, which further supports that HP-NAP directly and specifically interacts with TLR2. Thus, HP-NAP acts as a TLR2 agonist to activate neutrophils to secrete IL-8.

GPCRs that are expressed in neutrophils also participate in host defense and inflammation [18]. Most of these GPCRs couple to PTX-sensitive heterotrimeric Gi/o proteins and sense bacterial products, chemoattractants, or chemokines to mediate the activation and migration of neutrophils [18,35]. In this study, we show that PTX-sensitive heterotrimeric G proteins not only participate in the HP-NAP-induced ROS production, but also in the HP-NAP-induced, TLR2-dependent IL-8 secretion by neutrophils and the ATRA-induced differential HL-60 cells. Also, PTX-sensitive heterotrimeric G proteins are involved in the IL-8 secretion induced by Pam_3_CSK_4_, a TLR2 agonist, in ATRA-induced differential HL-60 cells. Consistent with our data, several studies reported that PTX-sensitive heterotrimeric Gi/o proteins modulate TLR2 signaling. For example, the Pam_3_CSK_4_-induced secretion of the C-C motif chemokine ligand 2 (CCL2) in human LAD2 mast cells was inhibited by the pretreatment with PTX, which is an inhibitor of Gi/o proteins [36]. The genetic depletion of Gαo protein in LAD2 cells also led to a reduction in the IL-8 secretion that was induced by Pam_3_CSK_4_ [37]. Furthermore, Pam_3_CSK_4_ is able to rapidly trigger the activation of heterotrimeric Go and Gi proteins [37,38]. Our data, together with the aforementioned findings, support the idea that PTX-sensitive heterotrimeric G proteins act downstream of TLR2 to mediate the HP-NAP-induced secretion of IL-8 by neutrophils. However, unlike Pam_3_CSK_4_, HP-NAP is an agonist of both TLR2 and a PTX-sensitive GPCR. The binding of HP-NAP to TLR2 could induce the interaction between TLR2 and the GPCR of HP-NAP by direct interaction with each other to mediate the IL-8 secretion by neutrophils. Such a unique interaction between two distinct innate immune receptors has been reported for TLR4 and protease-activated receptor 2 (PAR2), which is a GPCR [39]. In addition, we cannot exclude the possibility that the binding of HP-NAP to TLR2 and its GPCR activates two separate signaling pathways that converge to enhance the secretion of IL-8 by neutrophils. The precise molecular mechanism by which HP-NAP induces IL-8 secretion from neutrophils awaits further investigation.

In our study, the pretreatment of neutrophils with PTX only partially inhibited the production of ROS and secretion of IL-8 induced by HP-NAP (Figure 2A and Figure 7D), while the PTX pretreatment by itself induced neutrophils to secrete IL-8 (Figure 7D). PTX is a typical AB_5_ bacterial toxin composed of an A-protomer and a B-oligomer [40] and is well-characterized for its ability to catalyze the ADP-ribosylation of the α-subunit of Gi/o proteins [41]. The ADP-ribosylation that is mediated by the A-protomer of PTX further inhibits the activation of Gi/o proteins by preventing them from coupling to their cognate GPCR [42]. A previous study showed that PTX by itself was able to induce the secretion of IL-8 in endothelial cells through the activation of p38-MAPK [32]. The IL-8 secretion induced by PTX could be due to its effect on the ADP-ribosylation of Gi/o-proteins or its cellular actions that are independent of ADP-ribosylation. It has been reported that the PTX-mediated activation of p38-MAPK in the induction of the permeability of lung endothelial cells was dependent on the intrinsic ADP-ribosyl transferase activity of PTX [43]. Several ADP-ribosylation-independent actions of PTX have also been reported. Such actions include, for example, the binding of the B-oligomer to receptors such as TLR4 and platelet glycoprotein Ib at the cell-surface to induce various cellular responses [44,45], and the activation of tyrosine kinase, MAPK, and NF-κB [44,46,47]. Thus, PTX may simultaneously inhibit neutrophils to secrete IL-8 in response to HP-NAP by inhibiting the activation of Gi/o proteins and stimulate neutrophils to produce IL-8 by activating ADP-ribosylation-dependent and/or -independent pathways.

Both ATRA and DMSO induce the differentiation of HL-60 cells into neutrophil-like cells [22,23]. Our present study shows that only the differentiated HL-60 cells induced by ATRA, but not DMSO, were able to respond to HP-NAP to produce ROS. A previous study also showed that only the differentiated HL-60 cells induced by ATRA, but not DMSO, support the adherence of *E. coli* expressing opacity protein I (OpaI) [48]. It has been proposed that the induction of HL-60 cells to granulocytic maturation by ATRA and DMSO occurs via two different mechanisms [49]. Differential expression of the genes has also been observed in these two differentiated HL-60 cells [50,51]. The differences between ATRA- and DMSO-induced differentiated HL-60 cells in response to HP-NAP or *Escherichia coli* (*E. coli*) expressing OpaI may be due to the differential gene expression in these two differentiated cells. Our finding that HP-NAP-induced ROS production is initiated by the activation of a PTX-sensitive GPCR and followed by the activation of ERK1/2 and p38-MAPK in ATRA-induced differentiated HL-60 cells is consistent with the reports showing that the same signaling pathways are involved in the HP-NAP-induced ROS production in neutrophils [8,12]. Thus, the ATRA-induced differentiated HL-60 cells established here represent a suitable model for studying the neutrophil responses to HP-NAP and its underlying mechanisms.

Remarkable progress has been made in identifying the membrane receptors on neutrophils in response to their associated ligands and how these receptors regulate the functions of neutrophils. Here, we have shown for the first time that HP-NAP directly binds to and activates TLR2 to induce a PTX-sensitive heterotrimeric G protein-dependent secretion of IL-8 by neutrophils and ATRA-induced differentiated HL-60 cells. The PTX-sensitive heterotrimeric G proteins, but not TLR2, participate in the HP-NAP-induced ROS production by neutrophils and the differentiated HL-60 cells. Further studies are needed to address the question of how TLR2 and PTX-sensitive heterotrimeric G proteins are integrated to mediate HP-NAP-induced neutrophil responses. In addition, the ATRA-induced differentiated HL-60 cells, used here as neutrophil models, should provide a suitable approach to study the effects of HP-NAP on neutrophils. 

## 4. Materials & Methods

### 4.1. Cell Culture and Differentiation

The human promyelocytic HL-60 cell line [52] was cultured in RPMI 1640 medium (Cat. # 31800-014, Gibco, Thermo Fisher Scientific, Waltham, MA, USA) containing 20% heat-inactivated fetal clone III (Cat. # SH3010903, Hyclone, GE Healthcare Life Sciences, Little Chalfont, Buckinghamshire, UK) and 100 units/mL penicillin and 100 μg/mL streptomycin (Cat. # 15140-112, Gibco) at 37 °C with a humidified atmosphere containing 5% CO_2_. HL-60 cells were maintained at a density within the range of 1 × 10^5^ to 1 × 10^6^ cells/mL. The culture medium was renewed every two to three days.

HL-60 cells at an initial density of 3 × 10^5^ cells/mL in RPMI 1640 complete medium were added with 1 μM all-trans retinoic acid (ATRA, Cat. # R2625, Sigma-Aldrich, St. Louis, MO, USA) or 1.25% dimethyl sulfoxide (DMSO, Cat. # D2650, Sigma-Aldrich) and cultured for 4 days to induce the differentiation. Cell cultures were passaged by a 2/3 dilution two days after differentiation. The medium was renewed on day 3 and the cell density was adjusted to 6 × 10^5^ cells/mL. The differentiated HL-60 cells were ready for experiments on day 4. The morphological characteristics of the differentiated HL-60 cells were determined by microscopic examination of the Liu’s-stained cells on cytocentrifuged slides. The number of various types of myeloid cells was counted from 300 cells. The cell morphology and the percentage of each myeloid cell type present in the undifferentiated and differentiated HL-60 cells are shown in Supplementary Figure S1 and Table S1, respectively. The differentiated HL-60 cells are smaller in cell size and have multi-lobed nuclei with condensed chromatin (Figure S1). Banded and segmented nuclei were only found in the differentiated HL-60 cells. Spontaneous differentiation to mature myeloid cells occurred in 18% of the undifferentiated HL-60 cells, while the percentage of mature myeloid cells was increased to 86% in the ATRA-induced differentiated HL-60 cells and to 90% in the DMSO-induced differentiated HL-60 cells (Table S1).

### 4.2. Flow Cytometry

The extent of differentiation of HL-60 cells was determined by flow cytometry analysis of CD11b, the surface marker of neutrophils. Undifferentiated HL-60 cells and differentiated HL-60 cells with a cell number of 2 × 10^5^ were centrifuged at 600× *g* for 5 min, and then resuspended in 40 μL of fluorescence-activated cell sorting (FACS) buffer (Dulbecco’s phosphate-buffered saline (D-PBS), pH 7.2, containing 0.2% BSA and 0.1% sodium azide). The cells were either left unstained or stained with allophycocyanin (APC) mouse anti-human CD11b/Mac-1 (Clone ICRF44, Cat. # 561015, BD Biosciences, Franklin Lakes, NJ, USA) and APC mouse IgG_1_, kappa isotype antibodies (Clone MOPC-21, Cat. # 550854, BD Biosciences) by the addition of 10 μL of antibodies followed by the incubation on ice for 30 min. The cells were then centrifuged at 400× *g* for 5 min and washed once with 1 mL of FACS buffer. The cells were centrifuged again and then resuspended in 250 μL of FACS buffer. At least 10,000 cells were analyzed by BD Accuri^TM^ C6 flow cytometry (BD Biosciences) with an excitation laser at 633 nm and a 675/25 nm emission filter, detector FL-4. The percentage of CD11b-positive cells was determined by comparison of the cells that were stained with APC mouse anti-human CD11b/Mac-1 antibody and APC mouse IgG isotype antibody using BD Accuri C6 software from BD Bioscience. The percentage of CD11b positive cells was increased from 3.1% to 95.5% and 94.7% in undifferentiated HL-60 cells vs. ATRA-induced differentiated HL-60 cells and DMSO-induced differentiated HL-60 cells, respectively (Supplementary Figure S2).

### 4.3. Isolation of Human Neutrophils

Human neutrophils were isolated from peripheral blood that was collected with tubes containing sodium heparin by venipuncture from healthy adults. The blood was 1:1 diluted with Hanks’ Balanced Salt Solution (HBSS) without Ca^2+^, Mg^2+^ (Cat. # 14185, Gibco, Thermo Fisher Scientific) and followed by Ficoll-Paque PLUS (Cat. # 17144002, GE Healthcare Bio-Sciences AB, Uppsala, Sweden) density gradient centrifugation. Briefly, 25 mL of diluted blood was layered onto 12.5 mL of Ficoll-Paque PLUS and then centrifuged at 500× *g* for 30 min at 20 °C with brake off. The upper layer, which contained plasma, buffy coat and Ficoll-Paque PLUS, was discarded and the lower layer was diluted to 20 mL by HBSS and then subjected to dextran sedimentation by mixing with an equal volume of 3% dextran (Dextran 500, Cat. # 17-0320-02, Amersham Biosciences, Uppsala, Sweden) in 0.9% saline solution and followed by incubation at room temperature for 20 min. The leukocyte-rich fraction located at the upper layer was harvested and centrifuged at 300× *g* at 20 °C for 10 min. The pellet was subjected to two 30 s hypo-tonic treatments to remove the contaminated erythrocytes and then resuspended in RPMI containing 10% fetal bovine serum (FBS; SH3039603, Hyclone) and 2 mM L-glutamine or D-PBS, pH 7.2, containing 5 mM glucose (D-PBS-G). The viability of neutrophils was higher than 98% as determined by the trypan blue dye exclusion test. The purity of neutrophils was higher than 95% as determined by the morphological examination of at least 700 cells on cytocentrifuged slides stained with Liu’s stain using a Zeiss Axiovert 200 inverted microscope with 400 magnification (Carl Zeiss, Jena, Germany).

### 4.4. Expression and Purification of HP-NAP by Gel-Filtration Chromatography

The *napA* gene [Genbank: AE000543.1, Gene: HP0243] of *H. pylori* strain 26695 was cloned to the pET42a expression vector as described previously [10]. For the expression of recombinant HP-NAP, the *E. coli* BL21(DE3) strain carrying pET42a-NAP were grown in Lysogeny broth (LB) containing 50 μg/mL kanamycin (Cat. # 0408, Amresco, Solon, OH, USA) at 37 °C overnight. The fresh overnight culture with a volume of 2.5 mL was inoculated into the 250 mL of LB broth containing 50 μg/mL kanamycin and the bacteria were grown at 37 °C. After the optical density at 600 nm of the bacteria culture reached 0.4, the culture was added with 0.4 mM isopropyl β-D-1-thiogalactopyranoside (IPTG, Cat. # 101-357-93-1, MDbio, Taipei, Taiwan) and then incubated for 3 h at 37 °C until the optical density at 600 nm reached 1.6. The bacteria were harvested by centrifugation and washed once with D-PBS, pH 7.2. The cell pellet from the 120 mL culture was re-suspended in 6 mL of ice-cold D-PBS, pH 7.2, with the protease inhibitors as described previously [53]. The bacteria were disrupted by an ultrasonic processor SONOPLUS HD4200 with probe TS104 (BANDELIN, Heinrichstraße 3-4, Berlin, Germany) at 25% amplitude with 2 s ON/8 s OFF pulses for 30 min in a water bath with ice. Alternatively, the cell pellet from the 250 mL culture was resuspended in 20 mL of ice-cold D-PBS, pH 7.2, with the protease inhibitors and the bacteria were disrupted by a high-pressure homogenizer as previously described [53]. The bacterial lysates were centrifuged at 30,000× *g* at 4 °C for 1 h. For the purification of recombinant HP-NAP, 5 mL of the supernatant from the lysates was applied to gel-filtration chromatography using a XK 16/100 column packed with Sephacryl S-300 high resolution resin (Cat. # 17-0599-01, GE Healthcare Bio-Sciences AB) and the fractions enriched in HP-NAP were applied to another gel-filtration chromatography using a HiLoad 16/600 Superdex 200 prep-grade gel-filtration column (Cat. # 28-9893-35, Cytiva, Marlborough, MA, USA) as previously described [10]. The purified HP-NAP was passed through an Acrodisc unit with a mustang E membrane (Cat. # MSTG25E3, Pall, East Hills, NY, USA) to remove any endotoxin, adjusted to a concentration of 2 μM, and stored at 4 °C for no more than 2 weeks. The protein concentration was determined by the Bradford method using the Bio-Rad protein assay (Cat. # 500-0006, Bio-Rad, Hercules, CA, USA) with BSA as a standard. The purity of HP-NAP was higher than 95% as judged from sodium dodecyl sulfate-polyacrylamide gel electrophoresis (SDS-PAGE) analysis using a 15% polyacrylamide gel stained with Coomassie brilliant blue R.

### 4.5. Pretreatment with Inhibitors and Antibodies

ATRA-induced differentiated HL-60 cells and neutrophils were pretreated with 100 ng/mL PTX (Cat. # 516560, Calbiochem, Merck, Billerica, MA, USA) at 37 °C for 16 h and 4 h, respectively, to inhibit the activation of heterotrimeric Gi/o protein. ATRA-induced differentiated HL-60 cells were pretreated with 10 μM PD98059 (Cat. # A10705, AdooQ Bioscience, Irvine, CA, USA) and 5 μM SB202190 (Cat. # A10823, AdooQ Bioscience) at 37 °C for 1 h to inhibit the activation of MEK1/2 and p38-MAPK, respectively. For the blockade of TLR2 and TLR4, ATRA-induced differentiated HL-60 cells and neutrophils were pretreated with 10 μg/mL functional graded monoclonal mouse anti-human TLR2 (clone TL2.1) antibody (Cat. # 16-9922-82, eBioscience, San Diego, CA, USA), 10 μg/mL functional graded monoclonal mouse anti-human TLR4 (clone HTA125) antibody (Cat. # 16-9917-82, eBioscience), or 10 μg/mL functional graded mouse IgG_2a_, kappa monoclonal (clone eBM2a) isotype antibody (Cat. # 16-4724-85, eBioscience) at 37 °C for 30 min.

### 4.6. Measurement of ROS Production by NBT Reduction Assay

HL-60 cells or differentiated HL-60 cells in 50 μL of serum-free RPMI-1640 medium with a cell number of 2 × 10^5^ were added to 150 μL of D-PBS, pH 7.2, containing 0.13% (*w/v*) NBT (Cat. # N6495, Invitrogen, Carlsbad, CA, USA) and various stimuli, including PMA (Cat. # P1585, Sigma-Aldrich), N-formyl-methionyl-leucyl-phenylalanine (fMLP, Cat. # F3506, Sigma-Aldrich) and HP-NAP. The final concentration of PMA, fMLP, and HP-NAP were 0.32 μM, 1 μM, and 1 μM, respectively, unless otherwise specified. NBT was dissolved in water at a concentration of 2% (*w/v*) as the stock solution and its final concentration in the assay was 0.1% (*w/v*). The cell suspension, at a final volume of 200 μL, was incubated at 37 °C for 30 min and then placed on ice for 5 min to stop the reaction. Approximately 2 × 10^4^ of the cells were centrifuged at 600× *g* for 6 min onto a slide using a Shandon Cytospin 2 (Thermo Electron Corporation, Waltham, MA, USA). The slide was stained with Liu’s stain and the percentage of cells containing intracellular blue-black formazan was determined by counting at least 200 cells per slide under a light microscopy.

### 4.7. Measurement of ROS Production by DHE Fluorescence Assay

ATRA-induced differentiated HL-60 cells were resuspended in 125 μL of serum-free RPMI-1640 medium in the presence or absence of 4 μM DHE (Cat. # D7008, Sigma-Aldrich) at a density of 4 × 10^6^ cells/mL. The cells were then added to 375 μL of D-PBS, pH 7.2, containing various stimuli, including HP-NAP, Pam_3_CSK_4_ (Cat. # tlrl-pms, invivoGen, San Diego, CA, USA), or LPS (Cat. # tlrl-3pelps, invivoGen), and incubated at 37 °C for 30 min in the dark. The final concentrations of HP-NAP, Pam_3_CSK_4_, and LPS, were 1 μM, 1 μg/mL, and 10 μg/mL, respectively. The cells were centrifuged at 600× *g* for 5 min and washed with 1 mL of ice-cold D-PBS, pH 7.2. The cells were centrifuged again and resuspended in 250 μL of D-PBS, pH 7.2. DHE fluorescence was monitored by BD Accuri^TM^ C6 flow cytometry with an excitation laser at 488 nm and a 585/20 nM emission filter, detector FL-2. Data were analyzed with at least 30,000 cells per sample using BD Accuri C6 software from BD Bioscience.

### 4.8. Measurement of ROS Production by H_2_DCF-DA-Derived Fluorescence Assay

The production of intracellular ROS by neutrophils was measured by H_2_DCF-DA-derived fluorescence assay as previously described [54]. Briefly, human neutrophils in 50 μL of D-PBS-G at a density of 2 × 10^6^ cells/mL were added into individual flat-bottom wells of a 96-well black plate (Nunc, Rochester, NY, USA). Aliquots of 150 μL of D-PBS, pH 7.2, containing 13.4 μM H_2_DCF-DA (Cat. #. D399, Invitrogen, Vienna, Austria), 0.9 mM CaCl_2_, 0.5 mM MgCl_2_, and stimulus were added into each well. The final concentration of H_2_DCF-DA was 10 μM. The fluorescence emitted at 538 nm after excitation at 485 nm was monitored at 37 °C every 30 min for 3 h using a Wallac 1420-012 VICTOR 3 multilabel counter (Perkin-Elmer, Waltham, MA, USA). The level of ROS production was measured as an increase in the dichlorofluorescein (DCF) fluorescence intensity.

### 4.9. Immunoblotting

ATRA-induced differentiated HL-60 cells were suspended in D-PBS, pH 7.2, containing 10 mM glucose, 4 mM CaCl_2_, 4 mM MgCl_2_ at a density of 5 × 10^6^ cells/mL. After cell suspensions were added with an equal volume of 2 μM HP-NAP, the cells were incubated at 37 °C for 1 to 10 min and then immediately placed on ice to stop the stimulation. The cells were centrifuged at 2300× *g* at 4 °C for 5 min, washed once with 1 mL of ice-cold D-PBS, pH 7.2, and then harvested in 100 μL of the ice-cold modified radioimmunoprecipitation assay (RIPA) buffer containing 10 mM Tris-HCl, pH 7.4, 150 mM NaCl, 0.05% SDS, 1% sodium deoxycholate, 1% NP-40, 0.5 mM ethylenediaminetetraacetic acid (EDTA), 1 mM phenylmethanesulfonyl fluoride (PMSF), 1 μg/mL leupeptin, 10 μg/mL aprotinin, 1 mM Na_3_VO_4_, and 50 mM NaF. The cells were lysed by sonication on ice using an UP50H ultrasonic processor (Dr. Hielscher, Berlin, Germany) with an amplitude of 50% and 0.5 s per cycle for 30 cycles. Whole cell lysates were centrifuged at 16,100× *g* at 4 °C for 30 min. The concentration of soluble proteins present in the supernatant was measured by a BCA Assay kit (Cat. # 23225, Pierce, Thermo Fisher Scientific, Carlsbad, CA, USA). Equal amounts of soluble proteins were separated by SDS-PAGE with a 12% gel and then transferred to polyvinylidene difluoride (PVDF) membranes. Immunoblotting was performed as previously described [53] using the primary antibodies and secondary antibodies with the indicated dilution factors listed in Table 1. The pre-stained protein marker for immunoblotting was purchased from SMOBIO (Hsinchu, Taiwan). The protein bands were detected by enhanced chemiluminescence (ECL) kits (Western Lightning Plus-ECL, Cat. # NEL105001EA, PerkinElmer, Waltham, MA, USA; Immobilon Western Chemiluminescent HRP Substrate, Cat. # WBKLS0500, Millipore, Billerica, MA, USA) using the LAS-3000 imaging system (Fujifilm, Tokyo, Japan). The intensity of the band was quantified using the Multi GAUGE version 3.0 software (Fujifilm).

### 4.10. IL-8 Release Assay

ATRA-induced differentiated HL-60 cells were suspended in RPMI-1640 medium containing 10% fetal clone III, 4 mM L-glutamine, 100 units/mL penicillin, and 50 μg/mL streptomycin at a density of 2 × 10^6^ cells/mL. Human neutrophils were suspended in RPMI-1640 medium containing 10% FBS, 2 mM L-glutamine, 100 units/mL penicillin, and 50 μg/mL streptomycin at a density of 5 × 10^6^ cells/mL. Aliquots of 50 μL of suspension cells were added into individual wells of 96-well plates, and then 50 μL of D-PBS, pH 7.2, containing various stimuli, including HP-NAP, Pam_3_CSK_4_, and LPS, was added to each well. The final concentration of HP-NAP was 1 μM unless otherwise specified. The cells were incubated at 37 °C for 16 h. The cell suspensions were centrifuged at 600× *g* for 5 min. The amount of IL-8 present in the supernatant was measured by using the human IL-8 ELISA Ready-SET-Go kit (Cat. # 88-8086-88, eBioscience) or the human IL-8 uncoated ELISA kit (Cat. # 88-8086-88, Invitrogen, Vienna, Austria) according to the manufacturers’ instructions.

### 4.11. ELISA-Based Solid-Phase Binding Assay

MaxiSorp microplates (Cat. # 442404, Nunc) were coated with 0.05 µg of HP-NAP in 100 µL of carbonate-bicarbonate buffer, pH 9.6, at 4 °C overnight. The wells without coating were used as controls. All the wells were washed three times with 300 µL of PBS, pH 7.4, with 0.1% tween-20 (PBS-T) for 10 min each, blocked with 250 µL of PBS with 1% BSA for 2 h, and then washed three times with PBS-T. Recombinant human TLR2 protein Glu21-Leu590, with a C-terminal 10-His tag (TLR2-10xHis; Cat. # 2616-TR-050; R&D Systems, Minneapolis, MN, USA) and recombinant human TLR4 protein Glu24-Lys631, with a C-terminal 10-His tag (TLR4-10xHis; 1478-TR-050; R&D Systems) were added into individual wells of a HP-NAP-coated microplate at a range from 2.5 μg to 0.078125 μg in 100 µL of PBS-T with 1% BSA and the plate was incubated at room temperature for 2 h. After being washed once with PBS-T, the wells were incubated with 50 µg/mL of the mouse monoclonal anti-6x His tag antibody (GT359, Cat. # GTX628914, GeneTex, Hsinchu, Taiwan) or the mouse monoclonal IgG3 isotype antibody (B10, Cat. # GTX35027, GeneTex) in 100 µL of PBS-T with 1% BSA at 4 °C overnight. The wells were washed five times with 300 µL of PBS-T and then added with 100 µL of 25 µg/mL of the HRP-conjugated goat anti-mouse secondary antibody (Cat. # 115-035-003, Jackson ImmunoResearch, West Grove, PA, USA) and then incubated at room temperature for 1 h. The wells were washed five times with 300 µL of PBS-T and the binding of the recombinant TLR2 and TLR4 proteins to the immobilized HP-NAP was detected by the addition of 100 µL of 3,3’5,5’-tetramethylbenzidine (TMB) substrate (Cat. # 34028, Pierce, Thermo Fisher Scientific). After 10 min in the dark, the reaction was terminated by the addition of 100 µL of 2N H_2_SO_4_ and the absorbance at 450 nm was measured using an iMark microplate absorbance reader (Bio-rad).

### 4.12. Competitive ELISA Binding Assay

The competitive ELISA was performed by the pre-incubation of 0.625 μg of the recombinant human TLR2-10xHis protein in 100 µL of PBS-T together with either Pam_3_CSK4 or BSA (Cat. # A9647, Sigma-Aldrich), as a control, with an amount ranging from 50 μg to 0.78125 μg at room temperature for 1 h. Aliquots of the mixtures containing the recombinant human TLR2-10xHis protein and various concentrations of Pam_3_CSK_4_ or BSA were then added into individual wells of the MaxiSorp microplate pre-coated with 0.05 µg of HP-NAP and incubated at room temperature for 2 h. The rest of the steps were identical to those described in the ELISA-based solid-phase binding assay.

### 4.13. Statistical Analysis

Statistical analysis was performed by using Excel 2013 and Excel 2016 software. The data are expressed as mean ± standard deviation (SD). A paired, two-tailed Student’s *t*-test was used to analyze the statistical difference. *p* < 0.05 is considered as statistically significant.

## Figures and Tables

**Table 1:** Antibodies used in this study.

Name of the Antibody	Clonality	Host	Clone	Catalog Number	Concentration or Dilution Factor	Source
APC mouse anti-human CD11b/Mac1	Monoclonal	Mouse	ICRF44	561015	1:5 ^b^	BD Bioscience, Franklin Lakes, NJ, USA
APC mouse IgG1κ isotype control	Monoclonal	Mouse	MOPC-21	550854	1:5 ^b^	BD Bioscience, Franklin Lakes, NJ, USA
6x His tag	Monoclonal	Mouse	GT359	GTX628914	50 μg/mL ^e^	GeneTex, Hsinchu, Taiwan
mouse IgG2aκ isotype control	Monoclonal	Mouse	eBM2a	16-4724-85	10 μg/mL ^c^	eBioscience, San Diego, CA, USA
mouse IgG3 isotype control	Monoclonal	Mouse	B10	GTX35027	50 μg/mL ^e^	GeneTex, Hsinchu, Taiwan
p38α	Polyclonal	Rabbit	N/A ^a^	sc-535	1:1000 ^d^	Santa Cruz Biotechnology, Santa Cruz, CA, USA
peroxidase-conjugated goat anti-mouse IgG (H+L)	Polyclonal	Goat	N/A ^a^	115-035-003	1:5000 ^d^; 25 µg/mL ^e^	Jackson ImmunoResearch, West Grove, PA, USA
peroxidase-conjugated goat anti-rabbit IgG (H+L)	Polyclonal	Goat	N/A ^a^	111-035-003	1:5000 ^d^	Jackson ImmunoResearch, West Grove, PA, USA
phospho-ERK1/2	Monoclonal	Mouse	E-4	sc-7383	1:3000 ^d^	Santa Cruz Biotechnology, Santa Cruz, CA, USA
phospho-p38 MAPK (Thr180/Tyr182)	Monoclonal	Rabbit	D3F9	4511	1:1000 ^d^	Cell Signaling, Beverly, MA, USA
p44/42 MAPK (ERK1/2)	Antiserum	Rabbit	N/A ^a^	M5670	1:3000 ^d^	Sigma, St Louis, MO, USA
TLR2	Monoclonal	Mouse	TL2.1	16-9922-82	10 μg/mL ^c^	eBioscience, San Diego, CA, USA
TLR4	Monoclonal	Mouse	HTA125	16-9917-82	10 μg/mL ^c^	eBioscience, San Diego, CA, USA

^a^ N/A: Not available; ^b^ Flow cytometry; ^c^ Neutralization assay; ^d^ Immunoblotting; ^e^ ELISA.

## Data Availability

No new data were created or analyzed in this study. Data sharing is not applicable to this article.

## Supplementary Materials

The following are available online at https://www.mdpi.com/article/10.3390/ijms222111560/s1.
