# Supplementary material for: Helicobacter pylori Neutrophil-Activating Protein Directly Interacts with and Activates Toll-like Receptor 2 to Induce the Secretion of Interleukin-8 from Neutrophils and ATRA-Induced Differentiated HL-60 Cells
Source: Int J Mol Sci. 2021 Oct 26;22(21):11560. doi: 10.3390/ijms222111560 (PMC8584237; doi:10.3390/ijms222111560)
Supplement: Supplementary file 1 [file ijms-22-11560-s001.zip › ijms-1423258-supplementary.pdf]

Supplemental Table

Table S1. Differential counts of the differentiated HL-60 cells induced by ATRA or DMSO

Inducing agents[a]	Myeloid cell type, % of three hundred cells						
	myeloblast	promyelocyte	myelocyte	metamyelocyte	banded neutrophil	segmented neutrophil	mature myeloid cells[b]
None	15.6	66.3	13	5	0	0	18.0
1 µM ATRA	0.6	13	20.3	34.7	24	7.3	86.3
1.25% DMSO	0	10	22.7	46	15.7	5.7	90.1

[a]HL-60 cells were incubated with the indicated concentrations of inducing agents for 4 days.

[b]Mature myeloid cells include myelocytes, metamyelocytes, and banded and segmented neutrophils.

Supplemental Figures

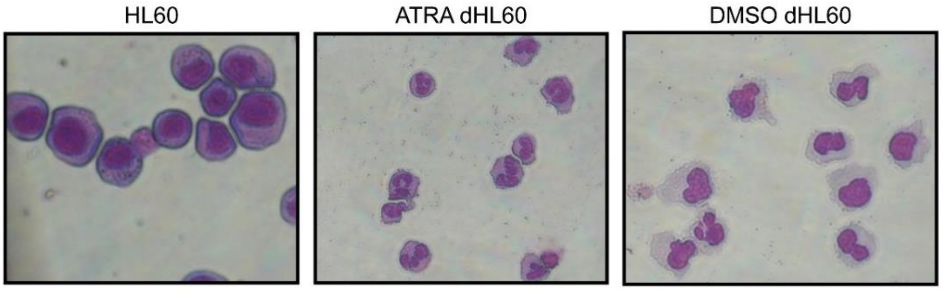

Figure S1. Changes in morphology of ATRA- and DMSO-induced differentiated HL-60 cells. HL-60 cells were seeded at a density of 3 x 10^5 cells/ml on day 0 and then incubated with 1 µM ATRA or 1.25 % DMSO for 4 days as described in Materials and Methods. Undifferentiated HL-60 cells and the differentiated HL-60 cells (dHL-60) were subjected to cytospin centrifugation and followed by Liu's staining. Cell morphology was observed under a microscope.

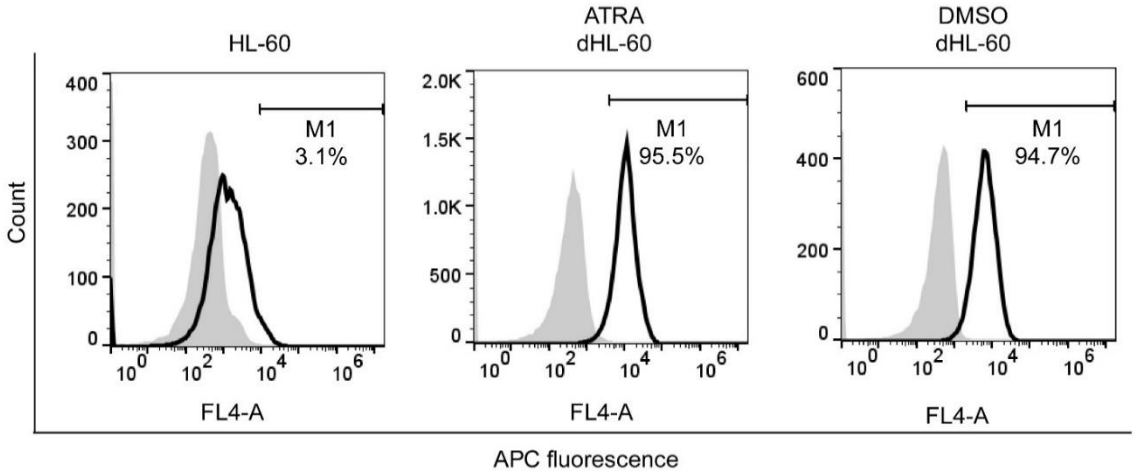

Figure S2. CD11b expression as a differentiation marker for ATRA- or DMSO-induced differentiated HL-60 cells. Undifferentiated HL-60 cells and the differentiated HL-60 cells (dHL-60) induced by ATRA or DMSO at a density of 1 x 10^6 cells/ml were stained with the APC-CD11b antibody (black open histograms) or the isotype control antibody (gray filled histograms) on ice for 30 min and were then subjected to flow cytometry analysis as described in Materials and Methods. The M1 region indicates the cell fraction being CD11b-positive. Data are representative of one to three independent experiments.
